# Nucleus high intensity in the T2-weighted MRI is a potential predictor of annulus tear in cervical injured patients: a case comparative study

**DOI:** 10.1186/s12891-023-06615-3

**Published:** 2023-07-24

**Authors:** Shengyu Wan, Jian Zhang, Chao Wu, Xu Lin, Jingchi Li, Fan Wu, Zifan Zhang, Lipeng He

**Affiliations:** 1 Department of Orthopaedics, Zigong Fourth People’s Hospital, Zigong, 643000 Sichuan Province People’s Republic of China; 2 Department of Orthopedics, The Affiliated Traditional Chinese Medicine Hospital of Southwest Medical University, No. 182, Chunhui Road, Luzhou, Sichuan Province 646000 People’s Republic of China; 3 Department of Spine Surgery, Changzheng Hospital Affiliated to the Naval Medical University, 200003 Shanghai, People’s Republic of China; 4 Department of Orthopaedics, Wuxi Hospital of Traditional Chinese Medicine, Wuxi, 214000 Jiangsu Province People’s Republic of China

**Keywords:** Nucleus high intensity, Annulus tears, Injured cervical patients, Independent predictors, surgicalSurgical indications

## Abstract

**Background:**

Segmental fusion operations assume paramount significance for individuals afflicted by full layers of annulus tears as they avert the perils of rapid disc degeneration and segmental instability. Structures with high signal intensity in the T2-weighted MRI can predict potential damage to the injured segment. Since local structures are shortly related biomechanically, this may be an effective predictor for annulus tears.

**Methods:**

A retrospective analysis of the clinical data of 57 patients afflicted by cervical injuries and subjected to single-segment ACDF has been performed in this study. The surgeon performed intraoperative exploration to assess the integration status of the annulus. The signal intensity of the prevertebral space, nucleus, and injured vertebral bodies were judged in the T2-weighted imaging data. Regression analyses identified independent predictors for annulus tears, and the area under the receiver operating characteristic curve (AUC) was computed to evaluate the predictive performance of potential independent predictors.

**Results:**

The occurrence of nucleus high intensity was significantly higher among individuals with annulus tears, and the nucleus high intensity was deemed an independent predictor for determining the presence of intraoperative visible annulus tears in patients with cervical injuries. AUC for nucleus high intensity was calculated as 0.717, with a corresponding p-value less than 0.05.

**Conclusions:**

In the realm of diagnosing annulus tears in injured cervical patients, nucleus high intensity in the T2-weighted MRI emerges as a promising predictive factor. Notably, this applies specifically to patients devoid of fracture and visible annulus tears in their MRI scans. Such positive outcomes should be regarded as prospective indications for ACDF.

## Introduction

Spinal trauma, a condition frequently encountered in clinical practice, presents a complex array of challenges. Internal fixation procedures become imperative to restore segmental stability in patients with spinal fractures [1, 2]. It is crucial to recognize that not all fracture-free patients can evade the need for surgical intervention [3]. For individuals afflicted by intervertebral disc (IVD) injuries, especially those marked by full layers of annulus tears, segmental fusion operations assume paramount significance as they avert the perils of rapid disc degeneration and segmental instability [4–7].

The existing array of imaging-based examination techniques, while extensively employed, fails to precisely diagnose annulus tears in patients who have suffered cervical injuries. Although dynamic X-ray radiography accurately detects segmental instability [8, 9], we must exercise caution in recommending this examination for injured cervical patients in order to prioritize their medical safety (after all, immobilization forms the cornerstone of trauma patient treatment). While magnetic resonance imaging (MRI) reveals partial annulus tears [10, 11], it comes with a disconcertingly high false negative rate and the attendant risk of missed diagnoses [12, 13].

Injured patients with spinal and joint afflictions typically undergo MRI scans as part of routine clinical practice. The T2-weighted imaging data within these scans reveal high intensity signals that accurately pinpoint local damage [14–16]. Notably, T2-weighted MRI commonly reveals high intensity in the vertebral bodies, prevertebral space, and nucleus of the injured segment among spinal trauma patients. From a biomechanical standpoint, it is imperative to recognize that stress concentration-induced damage does not confine itself to a specific structure [17, 18]. In essence, considering the biomechanical interplay between local structures, we propose that the aforementioned structures exhibiting heightened signal intensity within the injured segment might serve as potential prognosticators for annulus tears.

To substantiate this hypothesis, we conducted an in-depth review of clinical data on patients who underwent single segmental anterior cervical discectomy and fusion (ACDF). Through comprehensive analysis of patients' demographic and imaging data, we endeavored to determine the reliability of these indicators in diagnosing annulus tears identified intraoperatively. By identifying this topic, our findings carry theoretical implications for selecting surgical indications in cervical injured patients. To our knowledge, this study represents the first of its kind to illuminate this subject matter.

## Materials and methods

### Patient data collection

**Fig. 1:**
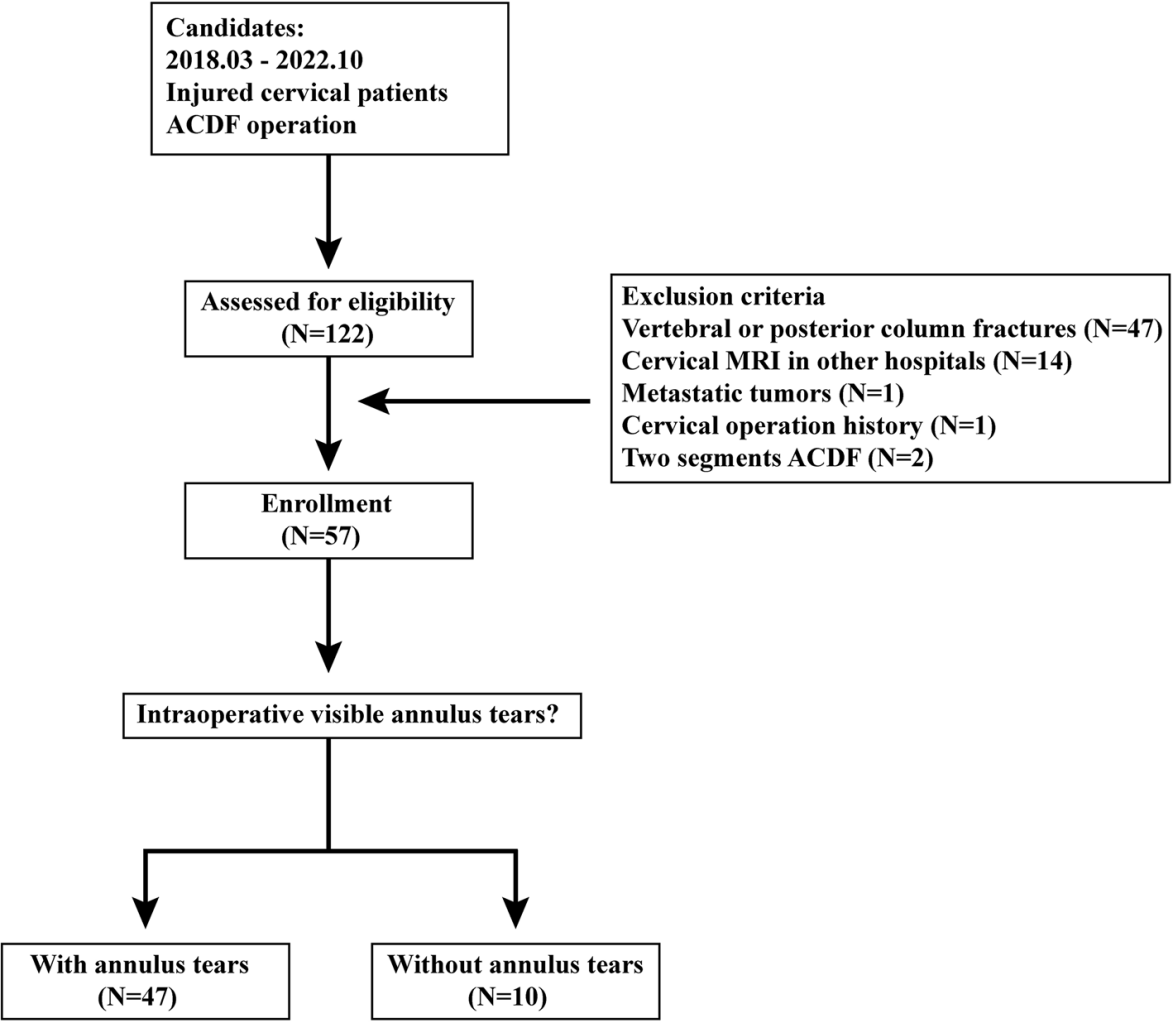
Schematic for patients inclusion and exclusion

Obtaining ethical approval from the ethics committees of our hospital served as the foundation for this study. Informed consent was waived due to the retrospective nature of the study [19, 20]. A retrospective analysis of the clinical data of 57 patients afflicted by cervical injuries and subjected to single-segment anterior cervical discectomy and fusion (ACDF) between March 2018 and October 2022 has been performed in this study. To ensure the integrity and relevance of our findings, specific inclusion and exclusion criteria were outlined. Patients meeting the inclusion criteria had to fulfill two primary conditions: firstly, they underwent single-level ACDF to address cervical injury and associated acute neurological symptoms, and secondly, they sought immediate medical attention at our hospital within three days of sustaining the injury and underwent a cervical MRI examination at our hospital's imaging department. In contrast, patients falling under the exclusion criteria were excluded from the study. These encompassed patients with cervical trauma accompanied by fractures or kyphotic deformity, individuals who underwent MRI examinations at other medical facilities, patients with a history of prior cervical operations, those suffering primary or metastatic spinal tumors, tuberculosis, or rheumatic immune diseases, as well as individuals who underwent multisegmental ACDF operations (Fig. 1). Concurrent with the imaging data, patients' demographic information including age, sex, and body mass index (BMI) was recorded and documented for comprehensive analysis.

### Intraoperative judgment of annulus tears and MRI-based parameters

The ACDF operation for all enrolled patients was conducted by a senior spine surgeon [21, 22]. The surgeon performed intraoperative exploration to assess the integration status of the annulus. Patients exhibiting visible full-layer annulus tears were thoughtfully categorized into the "annulus tears" group, while those demonstrating an intact outer-layer annulus found their place in the "without annulus tears" group. To ensure accuracy and consistency in our measurements, the assessment of MRI-based parameters was conducted using instant preoperative MRI data. A highly trained spine surgeon independently undertook the measurement of MRI-based imaging data.

**Fig. 2:**
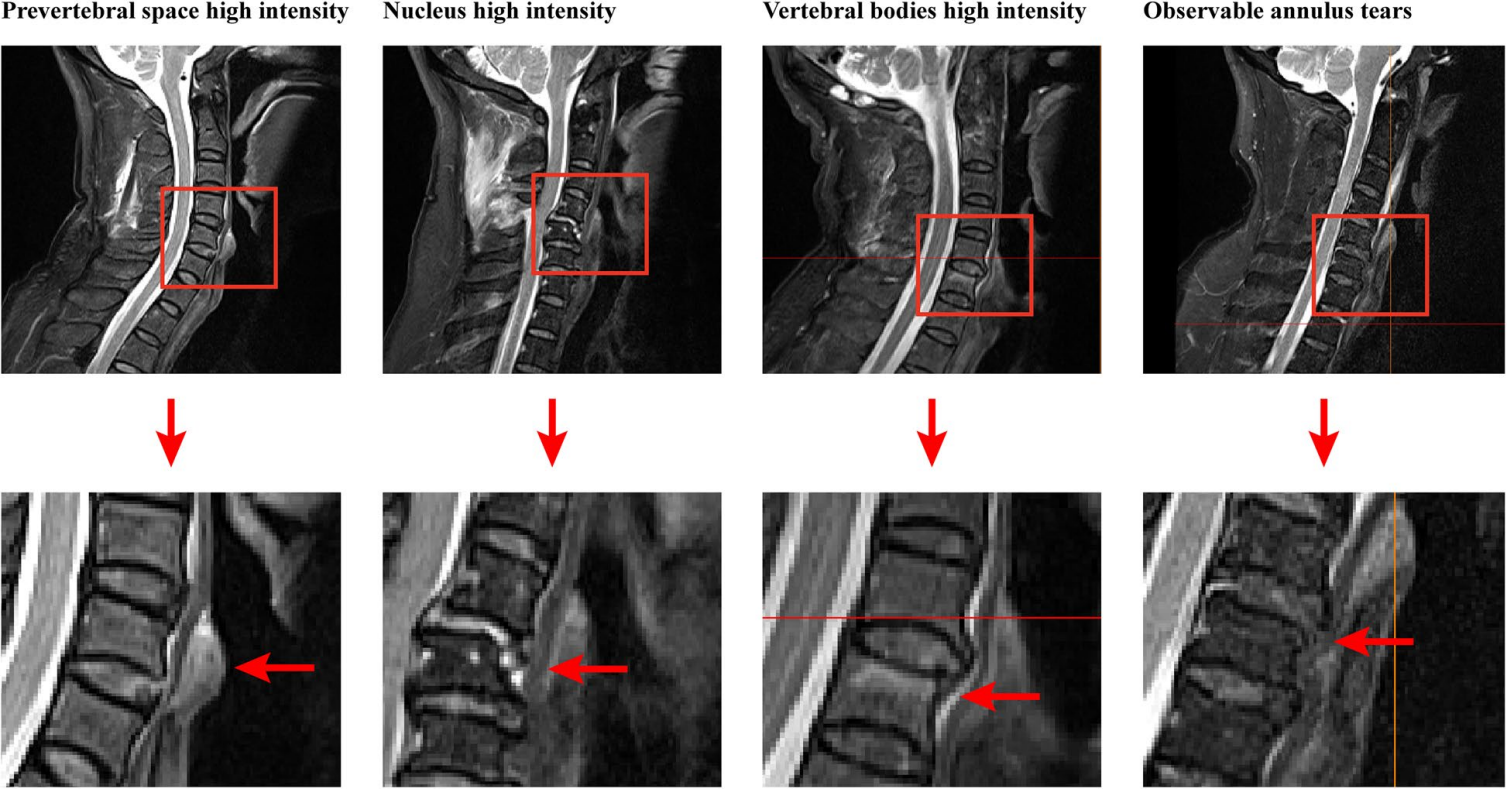
Schematic for MRI-based parameters

Within the T2-weighted imaging data, the signal intensity of various components served as a pivotal determinant. Since normal vertebral bodies typically exhibit low signals in T2-weighted imaging data, any vertebral bodies displaying heightened signal intensity were deemed indicative of injury. However, this particular definition method cannot be applied to judge nucleus damage since the normal nucleus tends to exhibit a high signal in this specific imaging sequence. Consequently, to ascertain the status of the nucleus—determining whether it exhibited an anomalously high signal intensity or not—a comparison was drawn between the signal intensity of the injured nucleus and that of adjacent segments. If the signal intensity of the nucleus appeared visibly higher than its neighboring segments, it was classified as injured [23, 24]. In our diligent quest for comprehensive understanding, the presence of prevertebral hematoma was defined by the presence of high-signal soft tissue in front of the injured vertebra. Additionally, we scrupulously assessed the integration of the annulus through meticulous scrutiny of the MRI scans. Any patients who presented observable annulus tears were meticulously recorded and documented for further analysis (Fig. 2) [13, 25].

### Statistical analyses

Under the expert guidance of a skilled statistician, the statistical analyses were diligently conducted using IBM SPSS 23.0 software. As a preliminary step, a normality test was performed to assess the distribution of continuous variables, such as patients' age and body mass index (BMI). Once it was established that these variables adhered to normality, they were expressed as mean ± standard deviation [26, 27]. On the other hand, binary variables, including sex, annulus integration status, and MRI-based parameters, were represented as percentages. An assessment was conducted to evaluate the reliability of measuring imaging parameters both within and between observers,. Ten randomly selected patients had their MRI-based parameters re-evaluated by both the spine surgeon and another surgeon from the same department. Since all MRI-based parameters were binary in nature, Kappa tests were employed to assess the consistency of measurement results among different groups. Kappa values ranging from 0.41 to 0.60 denoted moderate reliability, 0.61 to 0.80 indicated substantial agreement, and 0.81 to 1.00 reflected excellent or almost perfect agreement [19, 20, 28].

To compare the presence of demographic and MRI-based parameters between patients with and without annulus tears, distinct statistical methods were employed. Continuous variables were subjected to independent-sample Student's t-tests, while binary variables underwent chi-squared tests [29, 30]. Subsequently, a binary logistic regression analysis was conducted to identify the independent predictors for annulus tears. Univariate analyses were initially performed for each potential predictor, and variables that achieved a significance level of p-value < 0.1 were considered for subsequent multivariate analyses. Ultimately, variables with p-values < 0.05 in the multivariate analysis were identified as independent predictors for annulus tears [19, 20, 28]. In cases where the p-value of only one indicator fell below 0.1, an additional evaluation was conducted to ascertain if it was < 0.05 [31–33]. To assess the predictive value of potential independent predictors, ROC curve analyses were performed. The area under the receiver operating characteristic curve (AUC) was calculated to serve as an indicator of the predictive performance of MRI-based predictors. A p-value of less than 0.05 in ROC curve analyses signified that the corresponding parameter effectively predicted the presence of annulus tears [19, 20, 28].

## Results

### Data collection and significant differences between patients with and without annulus tears

During the designated period for clinical data collection, a comprehensive total of 122 patients underwent cervical surgery at our department to address cervical trauma. Among them, 47 patients were afflicted with either vertebral or posterior column fractures, while 11 patients had previously undergone cervical MRI examinations at other medical facilities. Notably, one patient received a diagnosis of metastatic tumors, another patient had a history of prior cervical operations, and two patients underwent multisegmental anterior cervical discectomy and fusion (ACDF) procedures. Consequently, a carefully selected cohort of 57 cervical injury patients (comprising 47 males and 10 females) who underwent single-level ACDF surgery, with an average age of 54.81 ± 11.17 years, were enrolled for this study. Upon analyzing the demographic data, no significant differences were observed between patients with and without annulus tears. The inter- and intraobserver reliability of measuring MRI-based parameters exhibited an acceptable level of agreement, and detailed kappa values, illustrating this reliability, are provided in Table 1.

**Table 1:** Kappa values of inter- and intraobserver reliability when measuring MRI based parameters

	**interobserver**	**intraobserver**
**Prevertebral soft tissues high intensity**	0.737	1
**Nucleus high intensity**	0.783	0.783
**Injured vertebral bodies high intensity**	1	0.8
**MRI observable annulus tears**	1	1

**Fig. 3:**
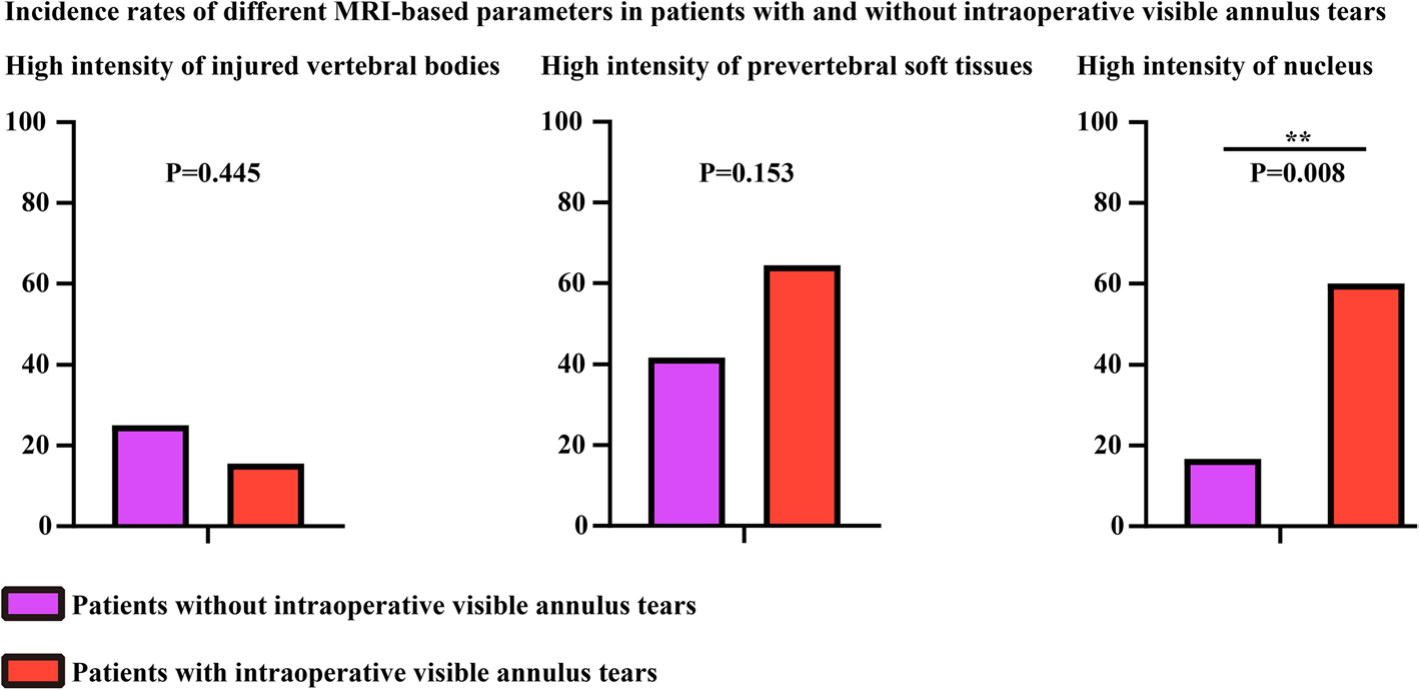
Differences in MRI-based parameters between patients with and without intraoperative visible annulus tears

Regarding the disparities in MRI-based parameters, it was discovered that the incidence rate of nucleus high intensity was 60% among patients with annulus tears, whereas it stood at 16.67% for patients without annulus tears. Consequently, the occurrence of nucleus high intensity was significantly higher among individuals with annulus tears. Conversely, the incidence rates of high intensity in the prevertebral space and injured vertebral bodies exhibited no significant discrepancies between patients with and without annulus tears. Notably, the rate of vertebral body high intensity was even insignificantly higher among patients without annulus tears. Furthermore, the incidence rate of observable annulus tears in the T2-weighted MRI scans reached 37.78% among patients with annulus tears, a figure significantly surpassing that of patients without annulus tears. None of the patients without annulus tears displayed any signals indicative of annulus tears in the MRI imaging data (Table. 2 and Fig. 3).

**Table 2:** Covariates for patients with and without annulus tear

	**Without annulus tear**	**With annulus tear**	***p-*value**
**Demographic covariates**			
Age	52 ± 7.14	55.56 ± 11.98	0.332
Sex (Male/Female)	10/2	37/8	0.928
BMI	23.38 ± 4.64	21.93 ± 2.55	0.155
**Potential predictor for annulus tear**			
High intensity of vertebral bodies in the injuried segment	3/9	7/38	0.445
High intensity of the nucleus	2/10	27/18	0.008**
High intensity of the prevertebral space	5/7	29/16	0.153
Observable annulus tear in the T2-weight imaging data	0/12	17/28	0.011*

^*^statistical significance in the multivariate regression analysis (*p-*value < 0.05)

### Potential predictors for annulus tears and the corresponding predictive performance

During the process of identifying potential predictors for annulus tears, it was observed that among the results of univariate logistic regression analyses, only the p-value associated with nucleus high intensity fell below 0.1. Consequently, a multivariate analysis was not conducted. Nevertheless, as the p-value for nucleus high intensity was less than 0.05 (p = 0.015), it was deemed an independent predictor for determining the presence of intraoperative visible annulus tears in patients with cervical injuries.

**Fig. 4:**
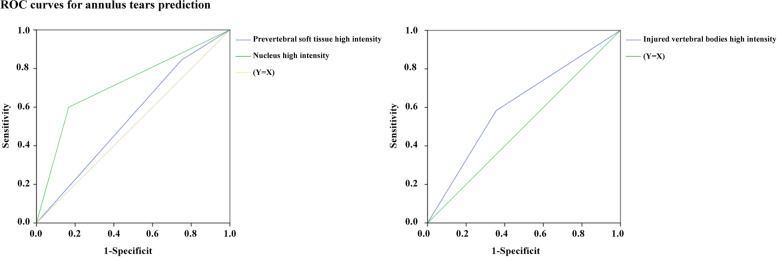
ROC curves of different MRI-based parameters

**Fig. 5:**
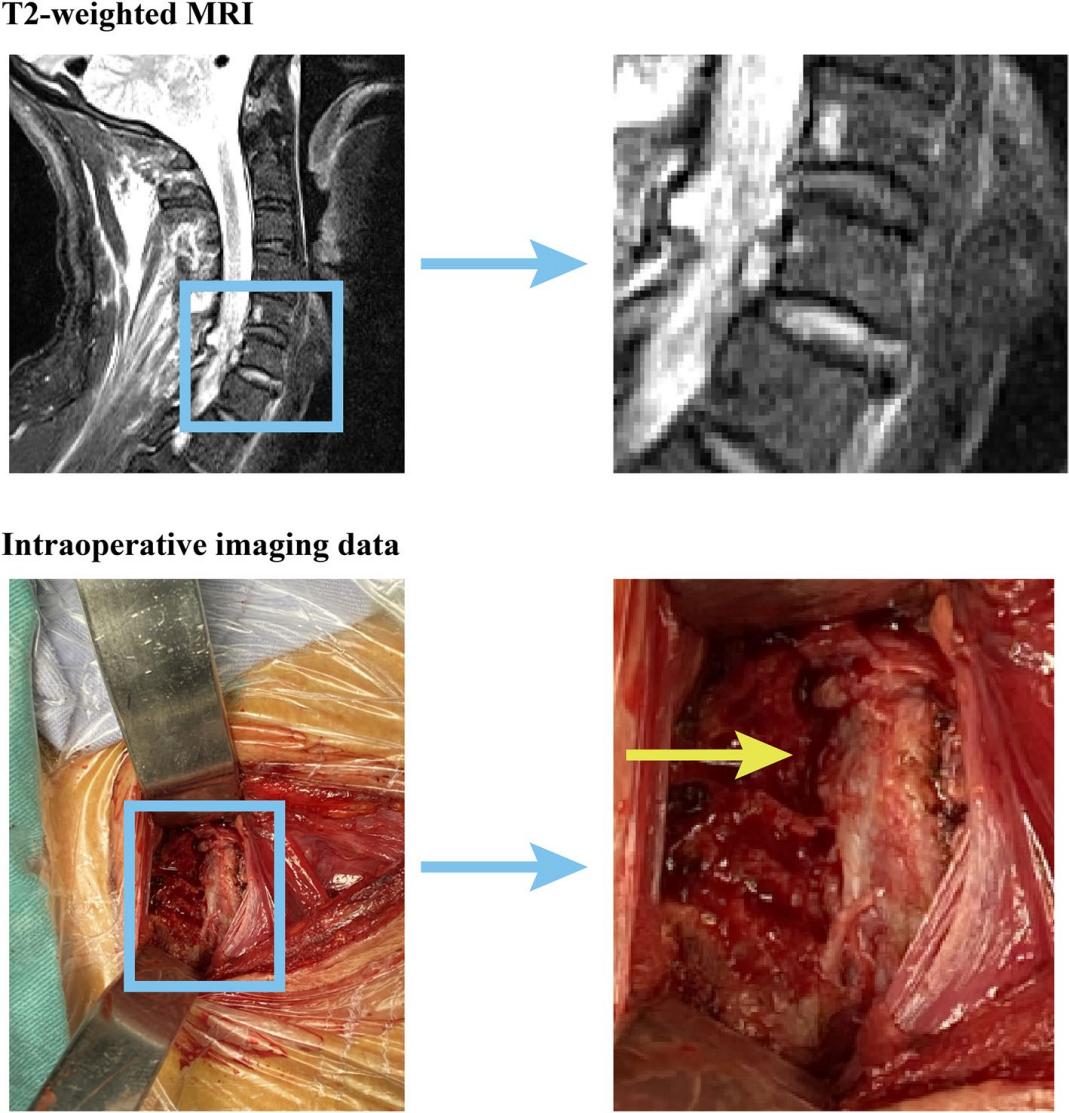
A typical case with nucleus high intensity and intraoperatively observed full laters annulus tear (Yellow arrow: intraoperative observed annulus tears)

To further evaluate the predictive value of nucleus high intensity, an ROC curve analysis was performed. The area under the curve (AUC) for nucleus high intensity was calculated as 0.717, with a corresponding p-value less than 0.05 (p = 0.022). In contrast, the AUC values for prevertebral space high intensity and vertebral body high intensity were determined as 0.547 and 0.641, respectively, with p-values exceeding 0.05. Detailed data pertaining to these analyses can be found in Tables 3 and 4, Figs. 4 and 5.

**Table 3:** Logistic regression analysis of annulus tear

	**OR**	**95% CI**		***p-*value**
**Univariate analyses**				
**Demographic variates**				
Age	0.972	0.918	1.029	0.329
Sex	0.925	0.169	5.062	0.982
BMI	1.165	0.943	1.44	0.157
**Potential predictor for annulus tear**				
High intensity of vertebral bodies in the injuried segment	0.553	0.119	2.566	0.449
High intensity of the nucleus	0.133	0.026	0.681	0.015*
High intensity of the prevertebral space	2.537	0.692	9.31	0.16
Observable annulus tear in the T2-weight imaging data	0.998	/	/	/

^#^variables that achieved a significance level of *p-*value < 0.1 in the univariate analysis

^*^statistical significance (*p-*value < 0.05)

^**^statistical significance (*p-*value < 0.01)

**Table 4:** The cut-off value, sensitivity and specificity for annulus tear’s prediction

	**Cut-off value**	**Sensitivity**	**Specificity**	**AUC**	***p-*value**
**High intensity of vertebral bodies**	0.5	0.583	0.644	0.614	0.229
**High intensity of the nucleus**	0.5	0.6	0.833	0.717	0.022*
**High intensity of the prevertebral space**	0.5	0.844	0.025	0.547	0.097

^*^statistical significance (*p-*value < 0.05)

## Discussion

The significance of the annulus, composed of multiple layers of fibers, has been extensively discussed in existing literature due to its biomechanical role in bearing tensile stress and maintaining segmental stability [34, 35]. Inflammation, a common pathological process in intervertebral disc (IVD) degeneration, is particularly relevant to annulus tears [17, 18, 36]. While the intact annulus prevents an immune response to the nucleus, the presence of annulus tears and subsequent capillary ingrowth trigger local inflammatory responses, leading to clinical symptoms [4, 10, 12]. Therefore, the effective identification of annulus tears is crucial to minimize misdiagnosis rates and ensure optimal clinical outcomes for patients [4, 13, 37–39]. However, diagnosing annulus tears in injured cervical patients poses challenges due to the limitations of available imaging examination methods. Dynamic X-ray radiography, considered the gold standard for assessing segmental range of motion and slide distances, cannot be performed on injured cervical patients due to the principle of immobilization in their management [40, 41]. T2-weighted imaging is commonly used to detect observable annulus tears, but its reliability as a parameter for annulus tear prediction is questionable due to a high false negative rate [10, 12, 13, 25].

Recently, a fiber tracking technique based on functional MRI examination has been proposed as an alternative method to predict annulus integration status [42]. However, this technique's effectiveness has yet to be extensively validated using large clinical samples, and its availability in primary healthcare institutions remains limited. Given that most cervical injury patients initially receive a diagnosis in primary hospitals, which are typically equipped with general MRI capabilities, exploring conventional MRI-based indicators for predicting annulus tears is of significant importance. This would help reduce the misdiagnosis rate of occult cervical injuries (without fractures or observable annulus tears in MRI) and optimize the clinical prognosis for these patients.

Motivated by the biomechanical integration of local structures and the correlation between T2-weighted high intensity and acute injury [5, 17, 18, 23, 24], this study proposes that high intensity in local structures could serve as a potential predictor for annulus tears. To validate this hypothesis, clinical data from injured cervical patients were collected, and the study confirmed that high intensity of the nucleus is an independent predictor for annulus tears. Based on these findings, we recommend that surgeons routinely assess the signal intensity of the nucleus in injured cervical patients. High nucleus intensity in occult cervical injuries should be considered an indication for anterior cervical discectomy and fusion (ACDF) to reduce the incidence of missed diagnoses in annulus tear patients and mitigate the potential risks of rapid disc degeneration and segmental instability. In contrast, high intensity in the prevertebral space and injured vertebral bodies could not effectively predict annulus tears in the corresponding segment. This suggests that soft tissue contusion in the prevertebral space does not necessarily indicate high stress or related injury to the annulus [5, 17]. Similarly, stress concentration in bony structures does not always coincide with concurrent IVD injury (as observed in patients with thoracolumbar and lumbar fractures but without injured IVD) [43, 44]. However, this study confirmed that damage and edema of the nucleus can effectively predict annulus tears, reinforcing the biomechanical relationship between these structures.

It is worth noting that this study lacks a control group receiving conservative treatment, and ACDF was performed on some patients without annulus tears. Dynamic radiography is strictly prohibited for patients with cervical spinal trauma to prevent severe consequences, making the evaluation of segmental stability or instability in MRI an indirect process. Considering that misdiagnosis of segmental instability can lead to significant prognostic deterioration, broader indications for ACDF have been chosen for patients with irregular signal intensity in T2-weighted MRI. Furthermore, injury is the primary cause of disc degeneration and segmental instability in medium to long-term follow-up periods. Therefore, even in patients without observable full-layer annulus tears, ACDF may still be an effective treatment strategy for cervical-injured patients to establish segmental stability and mitigate potential disc degeneration and segmental instability risks.While the main clinical significance of this study lies in presenting MRI-based parameters for more precise assessment of surgical indications in cervical-injured patients, there is a need to further investigate the prognosis of conservative treatment in patients without high nucleus intensity and observable annulus tears. In future studies, we will select this treatment strategy and report clinical follow-up results to reconfirm the conclusions drawn in this study and determine the effectiveness of conservative treatment in patients without observable full-layer annulus tears.

Admittedly, this study has inherent limitations. To validate the current results, larger sample sizes are necessary for verification. Additionally, the judgment of "high nucleus intensity" relies solely on the surgeon's subjective observation. Although intra- and interobserver reliability have been assessed through kappa analysis, a more objectively quantitative parameter should be developed to accurately evaluate the nucleus's signal intensity. Moreover, statistical analysis methods used in this study were commonly selected in the same type of studies [19, 20, 28]. Potential results of all possible sub-group analyses are ignored in the logistic regression analysis, and the random forest should be used in our future studies.

In summary, the annulus's biomechanical significance and its relation to inflammation and clinical symptoms have been well-documented. Diagnosing annulus tears in injured cervical patients using conventional imaging methods is challenging, and alternative techniques require further validation and wider availability. In this study, we propose that high intensity in local structures, particularly the nucleus, can serve as a potential predictor for annulus tears. The findings support the recommendation of routine assessment of nucleus signal intensity in injured cervical patients and consideration of high nucleus intensity as an indication for ACDF. However, future research with larger sample sizes and more objective quantitative parameters is warranted to validate these conclusions and improve the precision of annulus tear prediction in clinical practice.

## Conclusions

In the realm of diagnosing annulus tears in injured cervical patients, nucleus high intensity in the T2-weighted MRI emerges as a promising predictive factor. Notably, this applies specifically to patients devoid of fracture and visible annulus tears in their MRI scans. Such positive outcomes should be regarded as prospective indications for interbody fusion surgeries, as they hold the potential to mitigate the dangers of segmental instability and expeditious disc degeneration commonly associated with this particular patient cohort.

## Data Availability

All the data of the manuscript are presented in the paper.
